# Evaluation of three methods for betanin quantification in fruits from cacti

**DOI:** 10.1016/j.mex.2022.101746

**Published:** 2022-05-29

**Authors:** Luisaldo Sandate-Flores, Diana Valeria Rodríguez-Hernández, Magdalena Rostro-Alanis, Elda M. Melchor-Martínez, Carlos Brambila-Paz, Juan Eduardo Sosa-Hernández, Roberto Parra-Saldívar, José Rodríguez-Rodríguez, Hafiz M.N. Iqbal

**Affiliations:** a Tecnologico de Monterrey, School of Engineering and Sciences, Centro de Biotecnologia FEMSA, Avenida Eugenio Garza Sada 2501, Monterrey, NL, 64849 Mexico; b Laboratorio de Biotecnología, Facultad de Agronomía, Universidad Autónoma de Nuevo León, Francisco Villa S/N Col, Ex hacienda El Canadá, C.P. 66050 General Escobedo, N.L., México; c Tecnologico de Monterrey, Escuela de Gobierno y Transformacion Publica, Carlos Lazo 100, Colonia Santa Fe, Delegacion Alvaro Obregon, Ciudad de Mexico, 01389 Mexico

**Keywords:** Betanin, Molar extinction coefficient, Cactus fruits

## Abstract

In México, production of cacti fruits has increased. These fruits have high concentration of betalains (pigments), and market has increased interest in food with natural ingredients. In the near future, a sustainable method for betanin quantification in cacti fruits for rural communities will be necessary. Betanin in pulp of garambullo (Myrtillocactus geometrizan), chico fruit (Pachycereus weber), jiotilla (Escontria chiotilla) and pitaya de mayo (Stenocereus pruinosus) were quantified using three different analytical methods. The techniques were of Spectrophotometry UV-Vis (SCC), High-Performance Liquid Chromatography (HPLC) and Spectrophotometry technique using Molar Extinction Coefficient (SEC). The accuracy and intermediate precision were evaluated in SEC, SCC, and HPLC with the four cacti´s fruit. The means betanin concentration in the pulps were 0.68±0.05 (mg/g dry weight) garambullo, 1.28±0.06 (mg/g dry weight) chico fruit, 1.84±0.34 jiotilla and 2.0±0.25 pitaya de mayo (mg/g dry weight). The concentration of betanin in garambullo pulp measured by the three methods did not differ significantly (P >0.05). In this case, SEC method represents the best option to reduce costs, time and solvents in this way this method is aligned with green chemistry. In the three methods, coefficient of variation between measurements obtained are below 15%.

• *Robust method to quantify betanin and evaluate intermediate precision*
• *Validation parameters such as LOD, LOQ, accuracy, intermediate precision, and HorRat were considered*
• *The developed method enriches the valorization of underutilized national agricultural sources of Mexico*

## Graphical abstract

**Figure:**
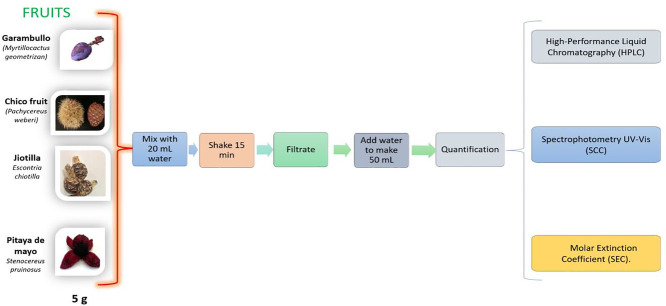

**Specifications table**

**Table:** 

Subject Area;	Environmental Science
More specific subject area;	*Environmentally related plants waste materials*
Method name;	*Spectrophotometry UV-Vis (SCC), High-performance liquid chromatography (HPLC) and* spectrophotometry using *molar extinction coefficient (SEC)*
Name and reference of original method;	*NA*
Resource availability;	***Chemicals,****Acetic acid >99.9% was procured from Sigma Aldrich, lot SHBD0354V, item 338826 500 ml St. Louis, MO. Acetonitrile 99.90 % was procured from TEDIA high purity solvents, lot 14090545, item AS1122-001, Fairfield OH. Betanin was procured ALDRICH chemistry, lot B00186845, item CD5000584-1G, Teutonia Wi. Water was obtained by a Milli-Q water purification system.****Experimental design and statistical analyses****, Results reported from 15 samples analyzed by three methods from each fruit were subjected to analyses of variance (α = 0.05) and Tukey test using MINITAB (Version 16.2.3, Minitab Inc., State College, PA Millipore Advantage A10, Billerica, Mass.*

## Data description – background

**Fig. 1:**
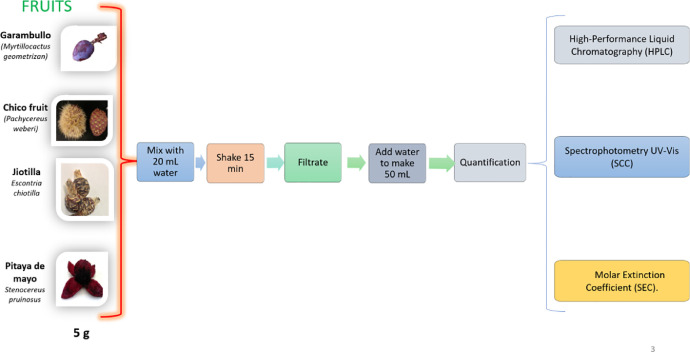
Scheme procedure of betalains quantification methods

Betalains are responsible for the color fruits such as prickly pear (*Opuntia*) [1], pitaya (*Stenocereus pruinosus*) [2], jiotilla (*Escontria chiotilla*) [3], and garambullo (*Myrtillocactus geometrizan*) [4]. In Mexico, the values of the production of pitaya (*Stenocereus pruinosus*) and prickly pear (*Opuntia*) has had a growth of 10.62% and 5.51% respectively in the last 3 years [5]. Furthermore, cacti juices have shown hepatoprotective [6], anticancer [7] and anticlastogenic effects [8]. With the benefit before mentioned, cacti fruits could be good resources of betalains. These compounds are divided in two groups betacyanins (red color) and betaxanthins (yellow color) [9]. Betanin (E162), main molecule in betacyanins, is internationally approved by FDA [10]. In the near future, a sustainable method for betanin quantification in cacti fruits will be necessary due to the increase of interest and production of these fruits. Analytical methods need to be compelling with the green chemistry. High performance liquid chromatography (HPLC) is a separation method as a highly quality technique due to the solvents used, although, signs of acute intoxication have been identified after accidental poisoning in occupational poisoning [11]. The spectrophotometric methods could be a solution to stop using solvents as long as there is no significant difference between both methods and the results have a good quality. The aim of this study is to stablish a methodology for quantification of betanin in pulp of garambullo (*Myrtillocactus geometrizan*), chico fruit (*Pachycereus weber*), jiotilla (*Escontria chiotilla*) and pitaya de mayo (*Stenocereus pruinosus)* based on green chemistry. The techniques of Spectrophotometry UV-Vis (SCC) and High-Performance Liquid Chromatography (HPLC UV-Vis at *λ=540 nm*) both with calibration curve and the spectrophotometry technique used the molar extinction coefficient (SEC) with non-calibration curve. Parameters of validation were evaluated in SEC, SCC, and HPLC with the four cacti´s fruit (Fig. 1).

## Method details

### Preparation of the cactus fruit crude extract

The batches of chico fruits (10 kg) and pitaya de mayo were collected from a crop field in Ahuatlan (between 18 ° 34′ latitude (North) and 98 ° 15′ longitude (West), Puebla, Mexico) and processed within 48 h of harvest. A batch of garambullo fruit from Myrtillocactus (3 kg) was procured of local market of Ahualulco (22° 24′ latitude (North) and 101° 10′ longitude (West) San Luis Potosi, Mexico) and processed within 24 h of harvest. A batch of jiotilla (22 kg) collected from a single crop field Santa Maria Zoquitlan, Tlacolula (16 ° 33′ latitude (North) and 96° 23′ longitude (West) Oaxaca, Mexico) were processed within 24 h of harvest. Fruit processing were carried out in Water Center of Tecnologico de Monterrey, Mexico. Fruits were washed with tap water and Extran MA05 (Merck, Item 1400001403, Lot Mx1400005004, Estado de Mexico, Mexico) and the prickles were manually removed. Peel and pulp were separated and the seeds were removed in a juice extractor (Model TU05, Turmix MR, Estado de Mexico, Mexico). Seed-free pulp samples were stored at -18 °C in 100 mL in bags of low-density polyethylene Ziploc® and used within 15 days after thawing at room temperature (25±2°C).

### Pigment extraction from fruit pulp

To evaluate the quantification methods SCC, HPLC and SEC, fifteen samples were prepared for each fruit. First, the pulp was defrosted, 5 g was weighed for each sample. The pulps were placed in a vial of 40 ml to which is added 10 mL of milli-Q water. Then, samples were agitated 15 minutes. After agitation, those were filtered through a paper filter (Whatman paper grade 4, 150 mm, Item 1009150, GE Healthcare Life Sciences, Little Chalfont, UK) under dark conditions to avoid betacianin degradation, then the filter was washed with 30 ml of milli-Q water and finally extracts obtained were filled to the mark 50 mL (Fig. 1). Moisture was determined by the gravimetric method in order to obtain analytes concentration in dry-weight [12].

### Calibration curves

Due to harvest time of the fruits, four stock solutions were prepared (betanin 0.5 g) and then this quantity was brought out to 10 ml with milli-Q water. The concentrations of solution stock were calculated with molar extinction coefficient (1120) [13]. The diluted solutions prepared for each calibration curve are shown in Table 1. A standard betacyanin linear calibration expression was used for betacyanin quantification (Eq. 1)

(1) $$BC=\frac{A_{c}+I_{c}}{S_{xc}}$$

where BC is the betacyanin concentration in the sample (mg/L), A_c_ is the absorbance at 540 nm, and I_c_ and S_xc_ are the intercept and slope of the betacyanin calibration curve. The calibration expression from each fruit are shown in Table 1.

**Table 1:** Betanin (*BC*) content in fruits (mg/g DW)

Pulp source	SEC	SCC	HPLC	Coefficient of variation (%)
*Myrtillocactus geometrizan* (garambullo)	0.69±0.0.05^a^	0.68±0.05^a^	0.68±0.04^a^	0.84
*Pachycereus weberi* (chico fruit)	1.31±0.06^a^	1.24±0.05^b^	1.30±0.06^a^	2.95
*Escontria chiotilla* (jiotilla)	1.97±0.29^a^	2.01±0.31^a^	1.54±0.22^b^	14.16
*Stenocereus pruinosus* (pitaya de mayo)	2.16±0.22^a^	2.09±0.21^a^	1.75±0.10^b^	10.97
*Stenocereus spp.*[14]	2.16±0.08^a^	2.04±0.08^a^	2.06±0.22^a^	3.08

### Chemical analysis

To carry out the chemical analysis the fruits were diluted in milli-Q water as follow: garambullo, jiotilla and chico extracts were diluted 2-fold, pitaya extracts were diluted 5-fold.

### Spectrophotometry UV-Vis (SCC) and High-Performance Liquid Chromatography (HPLC)

The diluted extracts obtained and the calibration curve were determined by a spectrophotometer (Model DR 500, Hach Lange GmbH, Düsseldorf, Germany) subsequently analyzed by liquid chromatography (Agilent Technologies, 1200 Series, Waldbronn, Germany) taking the absorbance at 540 nm [14]. The detector used in the HPLC was UV-Vis. Milli-Q water was used to dilute the samples. A column Eclipse XDBC18, 5 µm, 150 mm x 4.6 mm (Agilent Technologies, Santa Clara, CA) was used to perform the analysis. The mobile phase was: 85% v/v (A) water - acetic acid 1% v/v and 15% v/v (B) acetonitrile - acetic acid 1% v/v, the flow rate was 1 mL/min, column temperature was 25°C and the run time was 5 min.

### The molar extinction coefficient (SEC)

The following molar extinction coefficient was used, betacyanin (E_1%_ =60000 L mol^−1^ cm^−1^, λ=540 nm). The betacyanin concentration BC (mg/L) in the fruit's extracts were quantified by a spectrophotometer using the Eq. 1.

(1) $$BC=\frac{AxD_{f}xMWx1000}{E_{1\%}L}$$

Where E_1%_ is the betalains extinction coefficient, A is the absorbance at 540 nm, D_f_ is the dilution factor, MW is the molecular weights of betacyanin, L is the pathlength of the 1-cm cuvette.

### Validation method of SEC, SCC, HPLC in intermediate precision

#### Preparation of the samples for validation methods in reproducibility condition

**Fig. 2:**
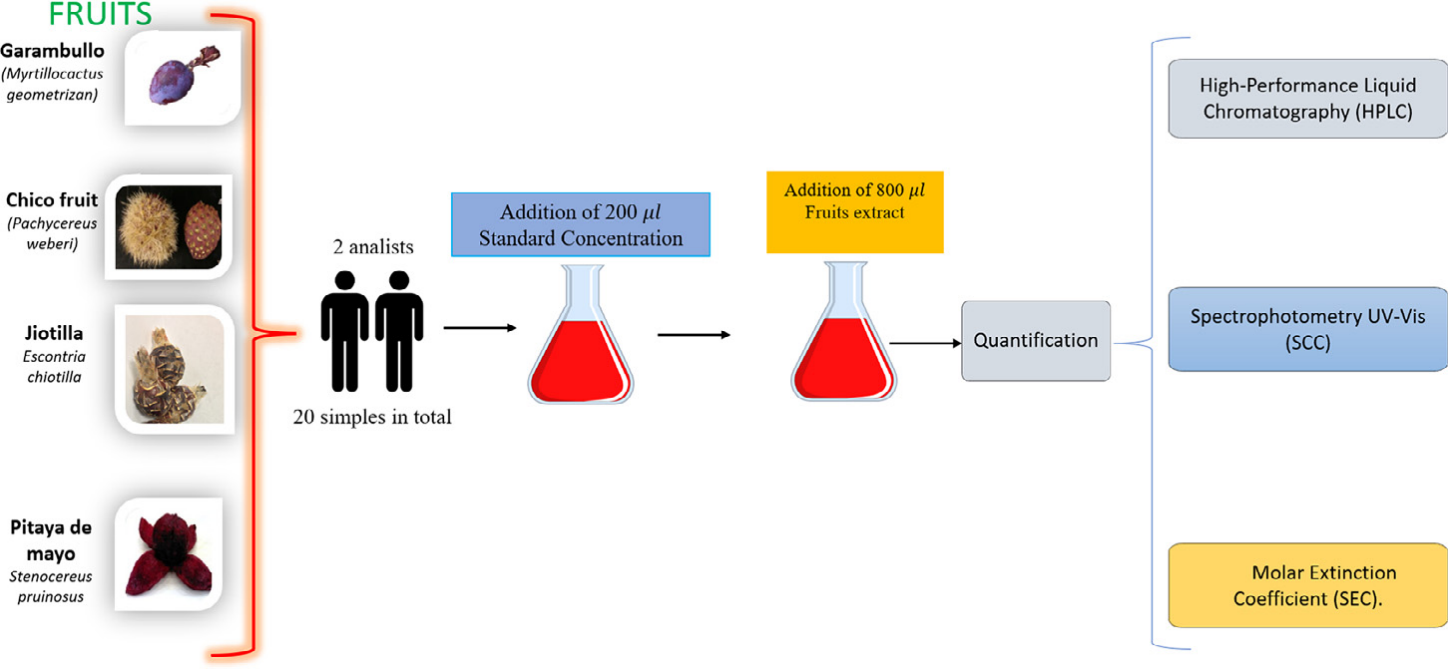
Scheme procedure to evaluate intermediate precision

Ten fortified samples were prepared for each fruit, five samples were prepared by analyst (1), and the rest of samples were prepared by analyst (2). The procedure was as follows: 0.2 mL of solution stock (known concentration) were added. Then, the volumetric flasks (1mL) were filled to the mark with cacti fruit´s extracts. The final solutions were passed to volumetric flasks (5 mL) and solutions were filled to the mark with milli-Q water (Fig. 2).

#### Accuracy

Accuracy (A) was calculated with the Eq. 2 where $\bar{X}$ is the mean of fortified concentrations in each method, and μ is true mean.

(2) $$A=\frac{\bar{X}-\mu }{\mu }X100$$

The true mean was calculated with the Eq. 3 where $C_{1}$ is the solution stock concentration, $V_{1}$ is the volume added of the solution stock (0.2 mL), $C_{2}$ is the fruit extract concentration, $V_{2}$ is the volume added of the fruit extract (0.8 mL), and $V_{f}$ is the volume final (5mL). The average of 10 replicates is the true mean.

(2) $$\mu =\frac{C_{1}V_{1}-C_{2}V_{2}}{V_{f}}$$

#### Precision (intermediate precision)

The precision (P) was obtained with Eq. 3, where (s) is the standard deviation and $\bar{X}$ is the concentration mean obtained by the two analysts.

(3) $$P=\left(\frac{s}{\bar{X}}\right)X100$$

#### HorRat value

HorRat value (H) is the ratio of the precision (P) calculated from the data to the predicted relative standard deviation (Pr) Eq. 4 [15]

(4) $$H=\left(\frac{P}{\bar{Pr}}\right)$$

Predicted relative standard deviation was calculated with Eq. 5, where C is expressed as a mass fraction [15]

(5) $$Pr=2C^{-0.15}$$

#### Limit of detection and limit of quantification

For SEC, SCC and HPLC methods, Limits of detection (LOD) were calculated using the Eq. 6, where s is the standard deviation of response. Limit of quantification (LOQ) was calculated with the Eq. 7 [16]

(6) LD=3s

(7) LQ=10s

**Experimental design and statistical analyses**, Results reported from 15 samples analyzed by three methods from each fruit were subjected to analyses of variance (α = 0.05) and Tukey test using MINITAB (Version 16.2.3, Minitab Inc., State College, PA Millipore Advantage A10, Billerica, Mass.

## Method results and discussion

The betanin concentrations in the cactus fruits analyzed were in the same order of magnitude. *Stenocereus pruinosus* pulp contains the highest concentrations of betalains (Table 1). Previous reports of betalains in cactus fruits such as *Myrtillocactus* using method SEC was in fresh weight (FW), in order to compare, the concentrations in the present study were changed to fresh weight. A higher concentration of betanin was founded in this work 131.81±10.05 mg/kg (FW) compared to 36.9 mg/kg (FW) reported by Guzmán-Maldonado et al. [17]. There are no reports of betanin quantification from the pulp of *Pachycereus weberii*, a lower concentration than pitaya pulp 2.86±0.38 mg/g DW by Garcia-Cruz et al. was determined [18] . The HPLC analysis of betanin from *Escontria chiotilla,* showed lower content than reported in the literature (2.2±0.01 mg/g DW) [3]. Betanin quantification from *Stenocereus pruinosus* was in agreement with the concentration reported by Sandate et al 2016 [14]. Meanwhile, garambullo pulp concentration was different from the values reported in the literature. Factors such as maturity, climate and, other factors could cause the differences in betanin concentration. The betanin concentration from garambullo measured by the three methods showed no significant difference in a similar way such as a previous report from pitaya analyzed by different methods (*Stenocereus* spp) [14].

The statistical analysis of the results using samples of pulps from *Pachycereus weberii, Escontria chiotilla* and *Stenocereus pruinosus* showed a statistically significant (*P* < 0.05) between the three different methods evaluated. Thus, an evaluation of the coefficient variation between the three methods was carried and a lower value than 15% in *Pachycereus weberii, Escontria chiotilla and Stenocereus pruinosus* was observed. Therefore, the selection of the method for betanin quantification could be dependent on the application and experimental stuff available. HPLC method is more specific than SCC and SEC due to the last methods quantify a wide range of similar compounds in a specific wavelength. However, SEC method is recommended due to the simplicity, the use of a solvent is reduced well-aligned with the principles of green chemistry (Table 2), non-specialized training is required and less time is necessary to perform the analysis. The SEC method could be used to evaluate the harvest index and postharvest quality in cactus fruits such as Ruiz Huerta et al has reported in jiotilla [19].

**Table 2:** Sustainability parameters of the methods.

Methods	Use of solvents	Time to performed the analysis	Equipment investment cost	Easy to performer the method
High-performance liquid chromatography (HPLC)	yes	High	High	Difficult
Spectrophotometry UV-vis (SCC)	No	Medium	Low	Easy
Molar extinction coefficient (SEC)	No	Low	Low	Easy

The methods were compared among them considering the application of rural community.

Table 3 showed results of the limit of detection (LOD), the limit of quantification (LOQ), accuracy and, intermediate precision. The betalains content in chico, jiotilla and, pitaya de mayo was higher than the limits of quantification, garambullo was unique below to the limit of quantification when the samples were analyzed by HPLC. The literature suggest that accuracy should be less than 15% [20], the analysis of garambullo sample with HPLC, chico sample with SEC and HPLC were more than 15%. HorRat is an assessment of the acceptability of the intermediate precision values, and it has been applied as one of the acceptability criteria for many adopted chemical analysis methods [21]. The acceptability range for HorRat is in a range of 0.5 to 2 [15]. The analysis of garambullo, chico, and jiotilla samples by HPLC was out the range. Pitaya de mayo had good intermediate precision from each method bases on HorRat value, intermediate precisión could be influenced by the concentration that the measuring equipment (Horwitz, Kamps, & Boyer, 1980) [22].

**Table 3:** Parameters of validation of methods

	Method	Limit of detectionmg/g DW	Limit of quantificationmg/g DW	Accuracy(%)	Intermediate precision(%)	HorRat value
*Myrtillocactus geometrizan* *(garambullo)*	SEC	0.2	0.68	14.74	10.31	1.60
	SCC	0.2	0.66	12.26	10.27	1.60
	HPLC	0.33	1.66	30.35	14.74	2.30
*Pachycereus weberi* *(chico fruit)*	SEC	0.19	0.64	21.38	5.15	0.92
	SCC	0.18	0.6	14.88	4.92	0.88
	HPLC	0.12	0.4	51.02	2.48	0.44
*Escontria chiotilla* *(jiotilla)*	SEC	0.22	0.72	6.82	4.87	0.72
	SCC	0.22	0.73	9.83	4.81	0.71
	HPLC	0.12	0.4	0.72	3.46	0.43
*Stenocereus pruinosus* *(pitaya de mayo)*	SEC	0.41	1.35	5.41	7.86	1.43
	SCC	0.38	1.26	2.32	7.56	1.37
	HPLC	0.47	1.55	-5.94	10.13	1.84

SCC is spectrophotometry UV-vis; HPLC is high-performance liquid chromatography; SEC is molar extinction coefficient; n=10. DW is dry-weight

SCC is spectrophotometry UV-Vis; HPLC is High-Performance Liquid Chromatography; SEC is molar extinction coefficient; Values represented as mean ± standard deviation (n = 15), different lowercase letters (a-b) indicate statistical significance differences (p<0.05). DW is dry-weight

## Conclusions

The quantification of betanin was determined in four samples from cactus fruits by SCC, HPLC, and SEC. SEC method is recommended as the best option to reduce costs, time and non-use of solvents for the sample of garambullo pulp, due to the betanin concentration have not significant differences between methods well-aligned with green chemistry principles. The coefficient of variation between methods showed values below 15%, evaluated in chico, jiotilla, and pitaya de mayo, this value could be an option to validate the application. HPLC method and SCC are not highly recommended because the equipment is expensive and for its handling requires highly specialized training, requires the preparation of chemical standards, and the evaluation of a calibration curve. Validation parameters such as LOD, LOQ, accuracy, intermediate precision, and HorRat were reported for each method.

## Declaration of Competing Interest

The authors declare that they have no known competing financial interests or personal relationships that could have appeared to influence the work reported in this paper.
